# PELP1 Is a Novel Therapeutic Target in Hepatocellular Carcinoma

**DOI:** 10.1158/2767-9764.CRC-24-0173

**Published:** 2024-10-07

**Authors:** Khaled Mohamed Nassar, Xue Yang, Adriana Baker, Rahul Gopalam, William C. Arnold, Timilehin T. Adeniran, Marian H. Hernandez Fernandez, Megharani Mahajan, Zhao Lai, Yidong Chen, Gangadhara R. Sareddy, Suryavathi Viswanadhapalli, Lu-Zhe Sun, Ratna K. Vadlamudi, Uday P. Pratap

**Affiliations:** 1 Department of Obstetrics and Gynecology, University of Texas Health San Antonio, San Antonio, Texas.; 2 Greehey Children’s Cancer Research Institute, University of Texas Health San Antonio, San Antonio, Texas.; 3 Department of Molecular Medicine, University of Texas Health San Antonio, San Antonio, Texas.; 4 Department of Population Health Sciences, University of Texas Health San Antonio, San Antonio, Texas.; 5 Department of Cell Systems & Anatomy, University of Texas Health San Antonio, San Antonio, Texas.; 6 Mays Cancer Center, University of Texas Health San Antonio, San Antonio, Texas.; 7 Audie L. Murphy South Texas Veterans Health Care System, San Antonio, Texas.

## Abstract

Hepatocellular carcinoma (HCC) is one of the leading causes of cancer-related deaths in the United States, with a median survival period of approximately 10 months. There is an urgent need for the development of effective targeted therapies for the treatment of HCC. Proline-, glutamic acid–, and leucine-rich protein 1 (PELP1) signaling is implicated in the progression of many cancers, although its specific contribution to the progression of HCC is not yet well understood. Analysis of The Cancer Genome Atlas HCC gene expression data sets and IHC analysis of HCC tissue microarray revealed that HCC tumors had elevated expression of PELP1 compared with normal tissues, and high expression of PELP1 is associated with unfavorable survival outcomes. Suppression of *PELP1* expression using short hairpin RNA significantly reduced the cell viability, clonogenicity, and invasion of HCC cells. Importantly, SMIP34, a first-in-class small-molecule inhibitor targeting PELP1, effectively decreased the cell viability, clonogenic survival, and invasiveness of HCC cells. Gene expression analysis using RNA sequencing revealed that *PELP1* knockdown cells exhibited a decrease in c-Myc, E2F, and other oncogenic pathways related to HCC. Mechanistic studies showed that SMIP34 treatment impaired the Rix complex, a critical component of ribosomal biogenesis, in HCC cells. Furthermore, the knockdown or pharmacologic inhibition of PELP1 significantly decelerated the HCC tumor growth in xenograft models. In summary, our study findings indicate that PELP1 could serve as a promising target for therapeutic intervention in HCC.

**Significance:**

HCC is one of the leading causes of cancer fatalities in the United States. Effective targeted therapeutics for HCC are urgently needed. In this study, we show that PELP1 proto-oncogene is crucial to HCC progression and that PELP1 inhibition reduced HCC cell proliferation *in vitro* and *in vivo*. Our results imply that PELP1-targeted drugs like SMIP34 may be useful as new therapeutic agents for HCC treatment.

## Introduction

Hepatocellular carcinoma (HCC) is the fourth leading cause of cancer-related deaths in the world and is the fastest growing cause of liver cancer deaths in the United States (1). Globally, liver cancer accounts for approximately 800,000 fatalities per year, and the global burden of liver cancer is estimated to be more than a million by 2025 (2). The prognosis of advanced HCC is exceedingly dismal, with a median survival rate of about 10 months (3). Furthermore, Latinos in South Texas have the highest liver cancer rates in the United States (4). Despite the execution of several surveillance programs to detect HCC at the earlier stages, most patients are diagnosed at advanced stages in which treatment options are limited (5). Thus, there is an unmet need to develop effective targeted therapies for the treatment of HCC.

Proline-, glutamic acid–, and leucine-rich protein 1 (PELP1) is a proto-oncogene that acts as a scaffolding protein in several chromatin remodeling and kinase signaling complexes (6). PELP1 is crucial in several biological pathways, such as nuclear receptor signaling, cell-cycle progression, and DNA damage response (7–9). The PELP1 protein possesses a Glu-rich domain that binds to histones, and it interacts with several complexes involved in altering chromatin dynamics (10–13). The oncogenic signaling of PELP1 is associated with the progression of various types of cancer, such as breast (14), endometrial (15), ovarian (16), salivary (17), prostate (18), lung (19), pancreas (20) and colon (21) cancers. Nevertheless, its contribution to the progression of HCC is still uncertain.

Recent studies suggested that PELP1 functions as a component of the Rix1 complex, which functions as a ribosome biogenesis regulator (22). Cryo-EM studies confirmed that PELP1 scaffolds the Rix1 complex, and its association with WDR18 may direct PELP1’s activity (23). Another study using PELP1 knockdown (PELP1-KD) model cells showed that PELP1-mediated oncogenic functions involve the regulation of several transcription factors, including c-Myc and ribosome biogenesis (24). Recent studies identified a small-molecule inhibitor, SMIP34, that binds PELP1 (25) and disrupts Rix1 complex function, leading to decreased protein synthesis (26). However, the therapeutic utility of SMIP34 in treating HCC is unknown.

This study focused on investigating the significance and therapeutic possibilities of targeting PELP1 in blocking the progression of HCC. Examination of The Cancer Genome Atlas (TCGA) HCC data sets and tissue arrays confirmed the increased expression of PELP1 in HCC. Suppression of PELP1, either through PELP1-KD or SMIP34 treatment, resulted in a decrease in cell viability, invasiveness, and clonogenicity of HCC cells. Mechanistic investigations demonstrated that PELP1 plays a crucial role in regulating the c-Myc and E2F pathways. Additionally, the KD of PELP1 or pharmacologic inhibition of PELP1 using SMIP34 significantly decreased the growth of HCC xenografts *in vivo*.

## Materials and Methods

### Cell lines and reagents

The human HCC cell line Huh7 (RRID: CVCL 0336) was a gift from Dr. Robert Lanford at the Texas Biomedical Research Institute in San Antonio, Texas. Other HCC cell lines (Hep3B, SNU398, SNU475, SNU423, and SNU449) were purchased from the ATCC and were maintained as per ATCC guidelines. The identity of cells was confirmed by short tandem repeat polymorphism analysis. All HCC cell lines were cultured in complete RPMI medium supplemented with 10% FBS and Antibiotic–Antimycotic. All the cell lines used in this study were free of *Mycoplasma* contamination. Synthesis of the PELP1 inhibitor SMIP34 was described in our earlier publication (25). PELP1-KD cells were generated using validated short hairpin RNAs (shRNA) and puromycin selection (1 μg/mL) using established protocol described in a previous publication (26). The p-mTOR (S2448), mTOR, p-S6 (S235/236), S6, p-STAT-3 (Y705), STAT3, p-Akt (S473), Akt, p-ERK (Thr202/Tyr204), ERK, p-4E-BP1 (Thr37/46), 4E-BP1, c-Myc, and GAPDH antibodies were obtained from Cell Signaling Technology. Beta-actin (A-2066) and vinculin antibodies (V9264) were purchased from Millipore Sigma. The Ki67 antibody (ab1667) was purchased from Abcam. The E2F1 (sc-251) antibody was purchased from Santa Cruz, and the GFP antibody (632381) was purchased from BD Biosciences. The WDR18 (15165-1-AP), TEX10 (17372-1-AP), and LAS1L (16010-1-AP) antibodies were obtained from Proteintech. Two PELP1 antibodies (A300-180A and IHC-00013) used in this study were purchased from Bethyl Laboratories, Inc. The GFP-PELP1 lentivirus construct was custom synthesized using VectorBuilder. Stable cells expressing GFP-PELP1 were generated by puromycin selection, and pooled clones were used for experiments.

### Cell viability, clonogenicity, and invasion assays

The cell viability rates of HCC cells expressing either *PELP1*-shRNA or treated with SMIP34 along with controls were determined by MTT (3-(4,5-Dimethylthiazol-2-yl)-2,5-Diphenyltetrazolium Bromide) assays (27). Clonogenic experiments were performed in 6-well plates, as described (27). Briefly, HCC cells expressing either control or *PELP1*-shRNA or cells exposed to vehicle or SMIP34 for a duration of 5 days were subjected to colony formation for a period of 14 days. Then cells were fixed with ice-cold methanol and stained using a 0.5% crystal violet solution. The colonies were quantitated using the NIH Image J program. The invasive potential of HCC cells expressing either *PELP1*-KD or treated with SMIP34 along with controls was evaluated using Corning BioCoat Matrigel Invasion Chamber assays using published protocol (27).

### RNA sequencing, RT‐qPCR, and Western blotting

Total RNA was isolated from control and *PELP1*-KD cells using RNeasy Mini Kit (Qiagen). The RNA sequencing (RNA-seq) analyses were conducted using the standard procedures of the Genome Sequencing Facility at the University of Texas Health San Antonio. The sequencing reads were mapped to the UCSC hg19 genome using the TopHat2 aligner, and the reads were quantified to the NCBI RefSeq genes using HTSeq. For the evaluation of functional enrichment pathways, the differential expression analysis conducted with DEseq2 utilized genes that exhibited a significant 2-fold change and adjusted *P* values below 0.05. Pathways were identified using gene set enrichment analysis (http://www.broadinstitute.org/gsea/index.jsp; ref. 28). A bubble plot was generated using online R-based visualization tool Hiplot (ORG; https://hiplot.org). The heatmaps of differential genes were produced using R software (version 4.2.1, R Core Team, 2022) and PHEATMAP package (version 1.0.12, https://cran.r-project.org/web/packages/pheatmap/index.html). RNA-seq data were deposited to the Gene Expression Omnibus database under the accession number GSE254860. Gene-specific primers were used to perform RT-qPCR to validate selectedgenes. The primer sequences are provided in the Supplementary Table S1. The RT-qPCR method was used as previously described (27). HCC cells were lysed using RIPA lysis buffer containing proteases and phosphatases inhibitors. Western blotting analysis was performed as described previously (27). Uncropped images of Western blots were included as Supplementary Fig. S2.

### Global protein synthesis analyses

For measuring global protein synthesis, HCC cells expressing either control shRNA or *PELP1-*shRNA or cells treated with SMIP34 (5, 10, and 15 μmol/L) for 16 hours were then treated with puromycin (Sigma-Aldrich, P7255; 10 μg/mL) for 30 minutes. Cycloheximide was used as a positive control as a blocker of protein synthesis. Total lysates were analyzed by Western blotting using an anti-puromycin (3RH11) antibody.

### Tissue microarray and IHC

Liver cancer tissue microarray (TMA; BC03119b) was purchased from TissueArray.Com (20855). The TMA contained 95 HCC and 10 normal liver tissues derived both from men and women patients. Some of the tissue cores were damaged; therefore, we only used good-quality cores for the analyses, which included 86 HCC and 6 normal liver tissues. IHC analysis of TMA using the PELP1 antibody was performed as previously described (26). IHC studies of mice xenograft tumors using Ki67 and PELP1 antibodies were conducted following established protocols (26). Five microscopic fields were randomly chosen and were used to calculate the percentage of Ki-67–positive proliferating cells. The intensity of PELP1 expression was quantified using Image J software.

### *In vivo* xenograft studies

All animal studies were conducted with the approval of the Institutional Animal Care and Use Committee at the University of Texas Health San Antonio. Female or male SCID mice, ages 8 to 10 weeks, were acquired from Charles River Laboratories. Hep3B cells stably expressing either the control vector or PELP1-KD (PELP1-shRNA1; 2 × 10^6^) were injected into the flank of SCID mice. A total of 6 to 8 tumors per group were used for xenograft experiments. For the SMIP34 treatment study, Hep3B xenograft tumor fragments measuring 2 to 3 mm^3^ were implanted subcutaneously on both flanks of female SCID mice. Treatment began once the tumor volume reached approximately 200 mm^3^. The control group was administered with vehicle, and the treatment group received SMIP34 (20 mg/kg/i.p./5 days a week) in 0.3% hydroxypropyl cellulose. The tumor’s volume was measured using a caliper at regular intervals of 3 to 4 days and calculated using a modified ellipsoidal formula. Tumor volume = 1/2(*L* × *W*^2^), in which *L* stands for the tumor’s longitudinal diameter and *W* for its transverse diameter. All mice were euthanized when the control tumors reached the size of ∼1,000 to 1,500 mm^3^, and their tumors were taken out and processed for histologic analysis.

### Databases and bioinformatic analysis

The *PELP1* gene expression in normal and HCC tissues was analyzed using TNMplot database analysis tool (https://tnmplot.com/; ref. 29). Survival analysis based on PELP1 protein expression was obtained from v23 (proteinatlas.org). PELP1 protein levels in normal and tumor samples in the Clinical Proteomic Tumor Analysis Consortium database was analyzed using the UALCAN (The University of ALabama at Birmingham CANcer data analysis Portal) website (30).

### Statistical analyses

GraphPad Prism 9 software was used for all statistical analyses (GraphPad Software). Statistics were compared between the control and *PELP1*-KD or vehicle- and SMIP34-treated groups using the Student *t* test and ANOVA. All the data in bar graphs are displayed as the mean ± SE. Significant results were defined as a *P* value less than 0.05.

### Data availability

All data supporting the conclusions are included in the manuscript and/or in the Supplementary Materials.

## Results

### PELP1 expression is upregulated in HCC and correlated with poor survival

**Figure 1:**
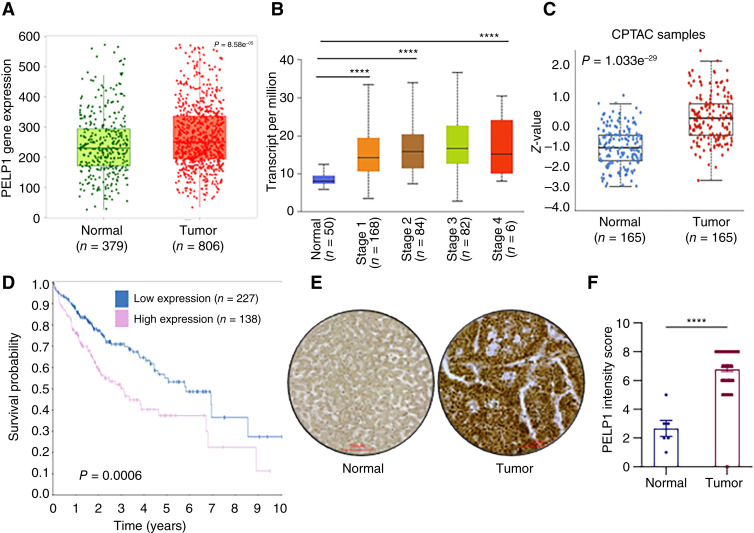
PELP1 expression is upregulated in HCC, and high PELP1 expression is associated with poor survival of patients with HCC. Data obtained from TNMplot shows increased expression of PELP1 in patients with HCC (**A**). The results from TCGA-UALCAN database show that PELP1 expression is increased with HCC progression (**B**). The Clinical Proteomic Tumor Analysis Consortium database shows high PELP1 expression in HCC tumor tissues compared with normal tissues (**C**). Association of protein expression of PELP1 with overall survival of patients with HCC was obtained from The Human Protein Atlas (**D**). TMA was used to investigate the PELP1 expression in 86 HCC and six normal liver specimens by IHC and quantified (**E** and **F**). Scale bar, 100 μm. *P* values are calculated using the Student *t* test. ****, *p* < 0.0001.

We utilized the TNMplot analysis tool, which enables the comparison of gene expression between tumor and normal tissues to examine alterations in *PELP1* levels in HCC. These results suggested that HCC tissues have a significantly higher level of PELP1 expression relative to normal liver tissues (Fig. 1A). The examination of the TCGA dataset utilizing the UALCAN database revealed a higher *PELP1* expression in all cancer stages compared with normal liver tissues (Fig. 1B). Analyses of data from the Clinical Proteomic Tumor Analysis Consortium using the UALCAN tool revealed that the expression of PELP1 is higher in HCC tumors compared with normal liver tissues (Fig. 1C). Further analysis of The Human Protein Atlas database also uncovered a negative correlation between the expression of PELP1 and survival of patients with HCC (Fig. 1D). We further validated the increased expression of PELP1 in HCC by IHC by using a TMA. The results indicated that the expression of PELP1 is increased in HCC tumors in comparison with normal liver tissues (Fig. 1E and F). Taken together, these results suggest that PELP1 is highly expressed in HCC and that its expression is associated with poor survival rates.

### PELP1-KD or SMIP34 treatment decreased the cell viability, clonogenic survival, and invasion of HCC cells

**Figure 2:**
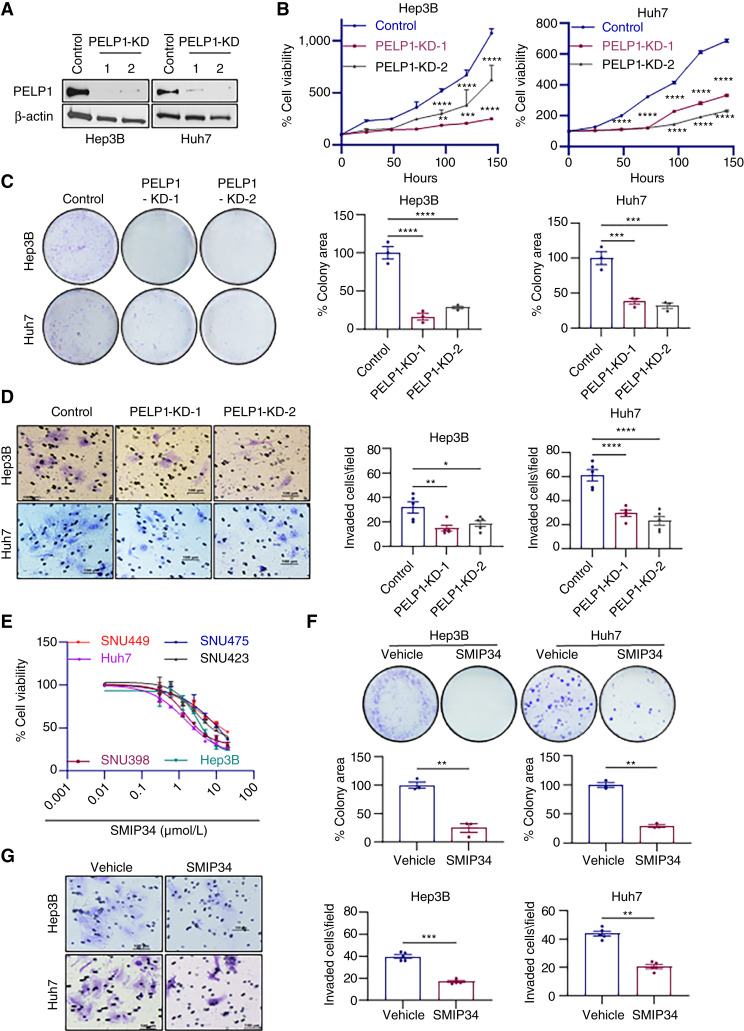
*PELP1*-KD/SMIP34 treatment decreased cell viability, clonogenicity, and invasiveness of HCC cells. PELP1-KD in Huh7 and Hep3B cell lines were confirmed by Western blot (**A**). Cell viability and clonogenic assays were performed to assess the impact of PELP1 KD on the growth of Huh7 and Hep3B cells (**B** and **C**). The effect of *PELP1*-KD on the invasion of HCC cells was performed using Matrigel invasion chamber assays (**D**). The effect of SMIP34 on the cell viability of six HCC cells was measured using MTT assay (**E**). Effect of SMIP34 (5 μmol/L) on clonogenic cell survival was examined using colony formation assay (**F**). The effect of SMIP34 (10 μmol/L) treatment on HCC cells invasion was performed using Matrigel invasion chamber assay (**G**). The results show the mean ± SEM, *n* = 3. *P* values are calculated using the Student *t* test and one-way ANOVA, *, *p* < 0.05; **, *p* < 0.01; ***, *p* < 0.001; ****, *p* < 0.0001.

To investigate the functional significance of PELP1 in HCC, we established *PELP1*-KD HCC cells using two distinct *PELP1*-shRNAs. Control non-target shRNA-transduced cells were used as controls. Western blotting analyses confirmed reduction in PELP1 levels in both PELP1-KD HCC cells (Fig. 2A). The MTT cell viability assay results showed that *PELP1*-KD significantly reduced the growth of both *PELP1*-shRNA–expressing cells compared with control HCC cells (Fig. 2B). Furthermore, *PELP1*-KD significantly reduced the clonogenic survival of HCC cells (Fig. 2C). Additionally, *PELP1*-KD resulted in reduced invasiveness of HCC cells compared with control cells (Fig. 2D). To further investigate the impact of PELP1 expression on the oncogenic capabilities of HCC cells, we augmented the expression of PELP1 in SNU475 and Hep3B cells which express relatively lower levels of PELP1 compared with other HCC cells by using a lentiviral vector (Supplementary Fig. S1A and S1B). The PELP1 overexpression models, specifically SNU475–PELP1-GFP and Hep3B–PELP1-GFP, demonstrated a 2-fold to 3-fold upregulation in PELP1 expression compared with the parental SNU475 and Hep3B cells (Supplementary Fig. S1B). Cell viability and clonogenic assays demonstrated that the upregulation of PELP1 significantly augmented the cell viability and clonogenic survival of HCC cells compared with the parental cells (Supplementary Fig. S1C and S1D).

Our group has recently identified SMIP34, a small-molecule inhibitor that binds and interferes with the oncogenic functions of PELP1 (25). We conducted MTT assays to examine the impact of suppressing PELP1 with SMIP34 on the viability of HCC cells. The findings from the experiment utilizing six different HCC cell lines indicate that SMIP34 is efficient in reducing the viability of HCC cells with an IC_50_ value of 1.5 to 15 μmol/L (Fig. 2E). Similarly, SMIP34 demonstrated efficacy in reducing the colony-forming capacity of HCC cells (Fig. 2F). In addition, treatment with SMIP34 resulted in a decrease in the invasion of HCC cells (Fig. 2G). Collectively, these findings suggest that PELP1 is necessary for HCC cell viability, survival, and invasion.

### PELP1-KD reduced the expression of genes associated with cell proliferation

**Figure 3:**
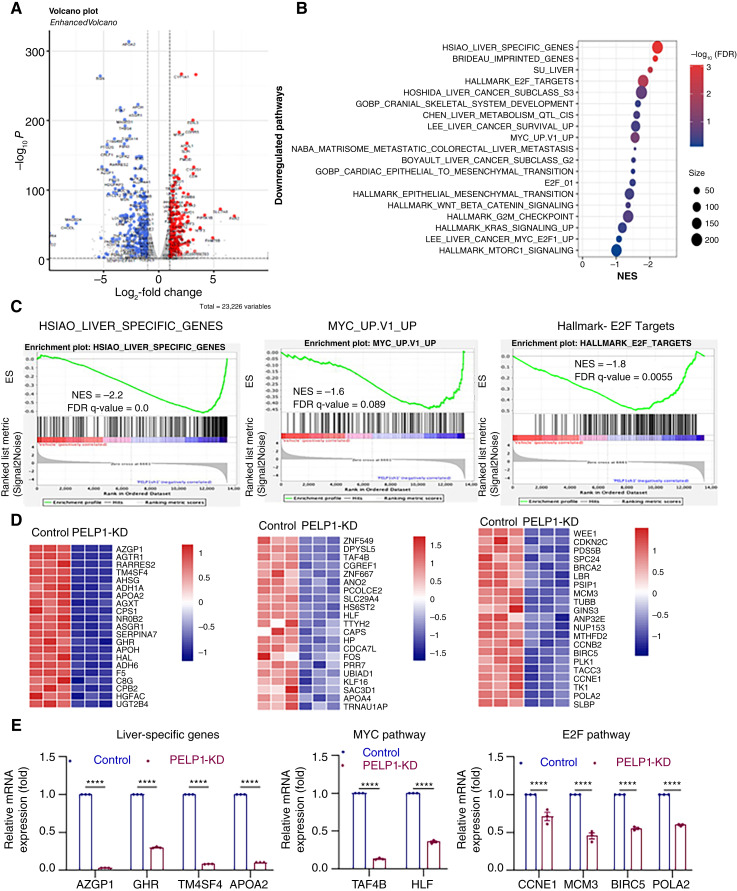
Analysis of global transcriptional changes in *PELP1*-KD HCC cells. Volcano plot of differentially expressed genes with PELP1-KD in Hep3B cells is displayed (*n* = 3; **A**). PELP1-downregulated pathways were identified using differentially expressed gene signature (**B**). Gene set enrichment analysis plots show negatively enriched pathways by PELP1-KD (**C**). Heatmap images of the specific genes affected by *PELP1*-KD (**D**). RT-qPCR was used to validate the specific genes that were differentially regulated by *PELP1*-KD in RNA-seq analysis (**E**). Data are represented as the mean ± SEM. *P* values are calculated using two-way ANOVA, ****, *p* < 0.0001. ES, enrichment score.

To comprehend the molecular mechanism(s) through which the inhibition of PELP1 decreases the viability of HCC cells, we examined the changes in gene expression in both control and PELP1-KD cells by RNA-seq. Overall, 592 genes (2-fold change over control cells with adjusted *P* value < 0.05) were differentially expressed in *PELP1*-KD cells compared with control cells. The volcano plot (Fig. 3A) illustrates the expression of top upregulated and downregulated genes between the control and PELP1-KD groups. The gene set enrichment analysis revealed a negative correlation between *PELP1*-KD–regulated genes and the *Myc*,* E2F*, and liver cancer–pathway specific genes (Fig. 3B–D). We used RT-qPCR to validate representative genes from each of the three pathways, and these results confirmed PELP1 regulation of these genes (Fig. 3E). Pharmacologic inhibition of PELP1 using SMIP34 also resulted in the downregulation of *Myc*, *E2F*, and liver cancer–specific genes (Supplementary Fig. S2A and S2B). These data suggest that PELP1 signaling has a substantial impact on reducing the expression of genes related to cell proliferation, such as *E2F* and *Myc*, in HCC cells.

### SMIP34 lowered PELP1 levels and affected PELP1 downstream kinase signaling

**Figure 4:**
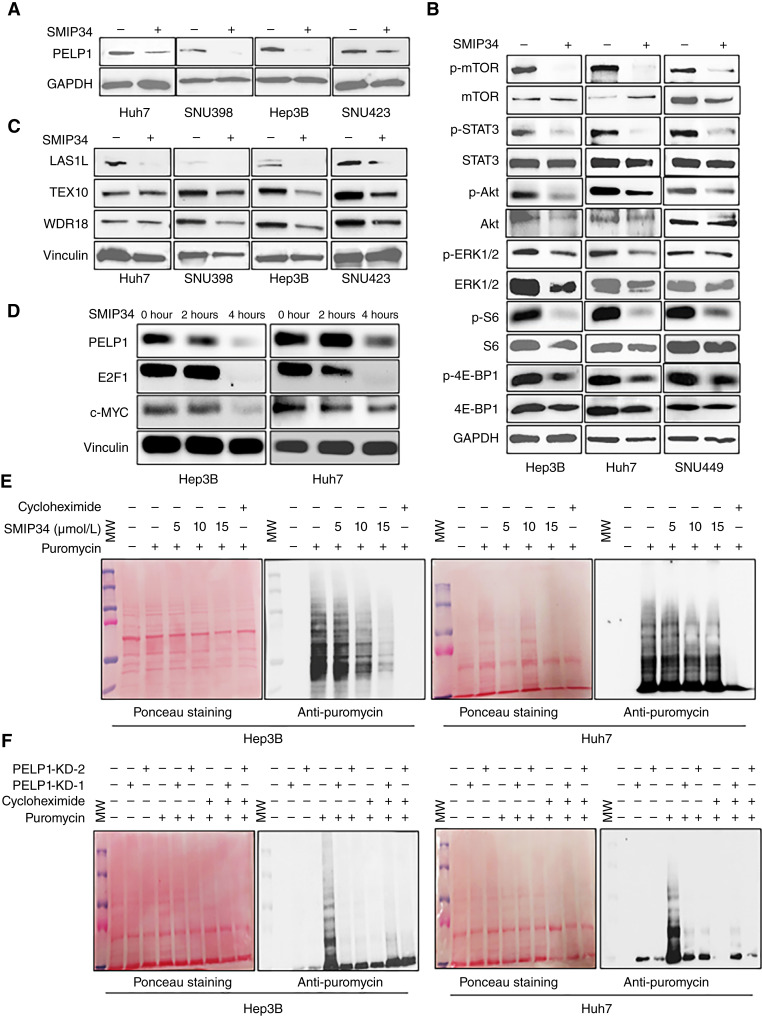
SMIP34 treatment blocked PELP1-mediated extranuclear signaling and decreased global protein synthesis. Huh7, SNU398, Hep3B, and SNU423 cells were treated with vehicle (DMSO 0.01%) or SMIP34 (12.5 μmol/L) to examine PELP1 degradation and the stability of the Rix1 complex by Western blotting (**A** and **C**). Hep3B, Huh7, and SNU449 cells were treated with vehicle (DMSO 0.01%) or SMIP34 (12.5 μmol/L), and the status of known PELP1 downstream signaling targets were analyzed by Western blotting (**B**). Hep3B and Huh7 cells were treated with vehicle (DMSO 0.01%) or SMIP34 (12.5 μmol/L), and the status of E2F1 and c-Myc expressions was analyzed by Western blotting (**D**). Hep3B and Huh7 cells treated with SMIP34 (5, 10, and 15 μm) or Hep3B and Huh7 cells with PELP1-KD were subjected to 30 miniutes of incubation with puromycin (1 μmol/L). The effect of this treatment on global protein synthesis was measured by Western blotting using an anti-puromycin antibody (**E** and **F**).

Prior research has shown that SMIP34 disrupts the process of PELP1 dimerization, resulting in reduced stability and the initiation of PELP1 degradation. We tested whether SMIP34 treatment induced the degradation of PELP1 in four different HCC cells by Western blotting. The findings demonstrated that treatment with SMIP34 substantially decreased the levels of PELP1 in HCC cells (Fig. 4A). Aside from its genomic roles, PELP1 is also involved in extranuclear signaling through direct interactions with Src-ERK, STAT3, and mTOR kinases (6). The Western blot analysis of HCC cell lysates, with or without SMIP34 treatment, revealed a significant reduction in PELP1 downstream signaling, including the phosphorylation of mTOR, STAT3, Akt, ERK, S6, and 4E-BP1 in the SMIP34-treated group as compared with the control group (Fig. 4B).

### SMIP34 treatment decreased Rix1 complex expression and protein synthesis

Previous investigations utilizing *PELP1* knockout cells and mass spectrometry analyses indicated that PELP1 plays a crucial role in maintaining the stability of the Rix1 complex (24). Consequently, we used Western blot analysis to assess the state of the Rix1 complex proteins in HCC cells in the presence of SMIP34 treatment. The Western blot analysis revealed that treatment with SMIP34 leads to a decrease in the amounts of WDR18/PELP1/Rix1 complex proteins WDR18, TEX10, and LAS1L in HCC cell lines (Fig. 4C). Earlier studies suggested that PELP1 regulates the expressions of c-Myc and E2F1. We, therefore, examined the expressions of c-Myc and E2F1 after a shorter SMIP34 treatment period in HCC cells. In both HCC cells, the levels of c-Myc and E2F1 were decreased within 2 to 4 hours following SMIP34 treatment (Fig. 4D). The alterations in the levels of c-Myc and E2F1 resulting from the pharmacologic inhibition of PELP1 for a shorter time period indicate that these fluctuations are not caused by growth inhibition, as the growth inhibitory effects mediated by *PELP1*-KD require a longer time.

Because PELP1 is an essential component of the Rix1 complex that facilitates the synthesis of new proteins, we investigated the impact of SMIP34 on new protein synthesis by treating HCC cells with different concentrations of SMIP34 and incubating them with puromycin for labeling of the C-terminus of translating polypeptide chains. New protein synthesis was measured by Western blotting analysis of whole lysates using an anti-puromycin antibody. The treatment of SMIP34 resulted in a notable reduction in overall protein synthesis in a dose-dependent manner (Fig. 4E). Cycloheximide treatment was used as a positive control, which effectively inhibited the production of new proteins. We also confirmed these findings by utilizing PELP1-KD HCC cells. The results demonstrated that PELP1-KD HCC model cells have significantly decreased amounts of new proteins compared with parental cells (Fig. 4F). Collectively, these findings indicate that PELP1 signaling in HCC cells plays a crucial role in the ribosomal processes regulated by the Rix1 complex and the synthesis of new proteins.

### PELP1 is essential for the growth of HCC xenografts *in vivo*

**Figure 5:**
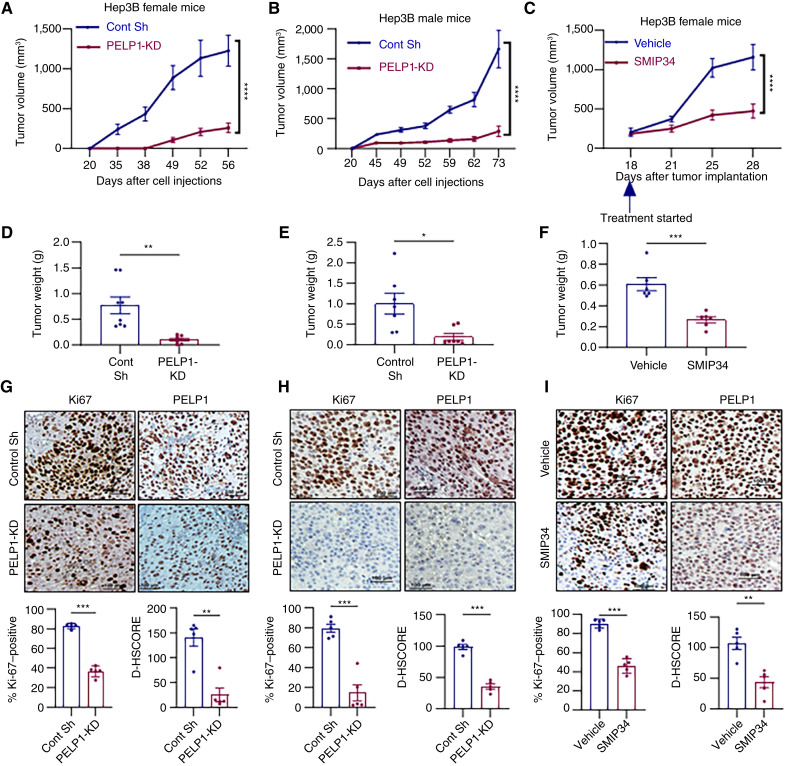
*PELP1*-KD or SMIP34 treatment suppresses HCC xenograft tumor growth *in vivo*. Hep3B–control and Hep3B–*PELP1*-KD model cells were injected subcutaneously into female (**A**, **D**, and **G**) or male SCID mice (**B**, **E**, and **H**). Tumor volumes were assessed at 3 to 5 days of intervals. Tumor volume (**A** and **B**), tumor weights (**D** and **E**), and IHC analyses of Ki67 and PELP1 (**G** and **H**) for tumors are shown. Hep3B xenograft tumor fragments measuring 2 to 3 mm^3^ were implanted subcutaneously , and following tumor establishment (∼200 mm^3^), the mice were randomly assigned to receive either vehicle (control) or SMIP34 (20 mg/kg body weight) 5 days a week through i.p. injection (*n* = 5). Tumor volume (**C**), tumor weights (**F**) and IHC analyses of Ki67 and PELP1 (**I**) for tumors are shown. Scale bar, 100 μm. Data are represented as the mean ± SEM. *P* values are calculated using Student *t* test and two-way ANOVA, *, *p* < 0.05; **, *p* < 0.01; ***, *p* < 0.001; ****, *p* < 0.0001. Cont sh (Control short hairpin).

In this study, we investigated the necessity of PELP1 for HCC growth *in vivo* by using xenografts of Hep3B model cells with or without *PELP1*-KD. Hep3B–control vector or Hep3B–*PELP1*-KD cells (2 × 10^6^) were injected subcutaneously into female SCID mice, and tumor growth was monitored. *PELP1*-KD in Hep3B cells led to a notable decrease in tumor volume and weights compared with control Hep3B xenografts, indicating that *PELP1*-KD hinders the progression of HCC tumors (Fig. 5A and D). IHC analyses utilizing Ki67, a marker of cellular proliferation, demonstrated decreased levels of proliferation in *PELP1*-KD tumors, along with decreased PELP1 expression in *PELP1*-KD xenografts, compared with controls (Fig. 5G). We also confirmed the impact of reducing PELP1 levels on HCC progression using xenografts in male mice. The results demonstrated that knockdown of PELP1 in Hep3B also greatly decreased the progression of xenografts as well as reduced levels of proliferation marker Ki67 and PELP1 in male mice (Fig. 5B, E, and H). We next evaluated whether pharmacologic inhibition of PELP1 using SMIP34 resulted in a reduction of tumor growth. Mice with Hep3B xenografts were randomized and administered with either vehicle or SMIP34 (20 mg/kg/i.p./5 days/week). SMIP34 treatment significantly reduced the tumor progression and tumor weight compared with vehicle-treated cells (Fig. 5C and F). Furthermore, IHC analyses revealed decreased levels of proliferation marker Ki67 along with decreased PELP1 expression in SMIP34-treated xenografts compared with controls (Fig. 5I). Overall, these findings indicate that PELP1 signaling plays a significant role in the growth of HCC xenografts.

## Discussion

More than a million people will be diagnosed with liver cancer by 2025, and about 90% of all liver cancer cases are HCC (3). Advances in basic and clinical research have helped in understanding the complex biology of HCC; however, it still remains as a very devastating disease. HCC diagnoses commonly occur in late stages, with drug resistance, clonal development, and tumor heterogeneity, contributing to reduced effectiveness of current therapies (31). Identification of molecular drivers that can be targeted for the treatment of advanced HCC is urgently needed. In this study, we identified that PELP1 is highly expressed in HCC and associated with poor survival rates. Using several HCC model cells, we provided evidence that PELP1 signaling plays a key role in the progression of HCC. Furthermore, PELP1 regulates c-Myc and E2F pathways and new protein synthesis. Finally, we demonstrated that PELP1 plays a critical role in HCC tumor progression *in vivo*. Collectively, these findings implicate a key role of PELP1 in HCC.

PELP1 has been recognized as a biomarker for cancer with negative prognoses, such as breast, prostate, ovarian, and endometrial cancers (32). Our analysis of TMA data revealed that the expression of PELP1 is increased in HCC relative to normal liver tissues. Moreover, the findings derived from publicly available databases corroborated the heightened expression of PELP1 in HCC. These findings suggest that dysregulation of PELP1 expression occurs in HCC. Nevertheless, future research using a greater number of HCC tumors is needed to validate these findings and to establish PELP1 both as a prognostic biomarker and a target for therapeutic intervention in HCC. Earlier studies have shown that *PELP1*-KD reduced cell growth, survival, and migration in endometrial cancer cells (26, 33). Corroborating these findings, using *PELP1*-KD model cells, we demonstrated that PELP1 is essential for cell viability, survival, and invasion in HCC.

The oncogenic signaling of PELP1 is established in several cancers, such as breast, endometrial, ovarian, salivary, prostate, lung, pancreatic, and colon cancers (15–21). However, its role in HCC remains unknown. Wnt-β-catenin signaling pathway activation is found in 30% to 50% of HCC cases due to mutations in β-catenin, AXIN1, or APC (34). Furthermore, epigenetic regulation, oxidative stress, and the Akt–mTOR and MAPK pathways are also linked to HCC (35). Additionally, CCND1, FGF19, VEGFA, and MYC chromosomal amplifications activate oncogenic signaling pathways (36). Earlier studies implicated PELP1 signaling in the activation of multiple oncogenic pathways, including the Wnt-β-catenin signaling, Akt–mTOR, and cMyc pathways (6). The results from RNA-seq studies indeed confirmed that *PELP1*-KD significantly reduced the expression of genes involved in E2F, epithelial–mesenchymal transition, β-catenin, Ras, and c-Myc pathways.

A recent study utilizing inducible *PELP1* knockout showed that PELP1-mediated oncogenic activities involve the regulation of several transcription factors, including c-Myc, as well as ribosome biogenesis (24). Through RNA-seq–based research, we have found that PELP1 plays a role in regulating the c-Myc pathway in HCC cells. The PELP1-TEX10-WDR18 complex serves as a regulator of ribosomal biogenesis and controls the pace of ribosomal synthesis (22). By manipulating the recruitment and release of the AAA-ATPase MDN1, PELP1 functions as a regulatory checkpoint in the maturation of the mammalian 60S ribosomal subunit (37). Our findings using pharmacologic inhibition of PELP1 revealed a significant reduction in the levels of Rix1 complex proteins, including WDR18, TEX10, and LAS1L. Our investigations into the mechanisms using puromycin labeling experiments have validated that the administration of SMIP34 or the KD of PELP1 leads to a reduction in protein production in HCC cells. Collectively, these findings suggest that the SMIP34-mediated growth inhibitory effect is in part mediated by blocking PELP1-facilitated functions in ribosomal biogenesis and protein synthesis.

Recent research has demonstrated the efficacy of the PELP1 inhibitor SMIP34 (25). Mechanistic investigations demonstrated that SMIP34 interferes with the formation of PELP1 homodimers and diminishes the dimerization of PELP1, leading to a decrease in the stability of PELP1 (26). The utility of SMIP34 as a PELP1 inhibitor has been validated in multiple cancer models, including estrogen receptor^+^ breast cancer (25), triple-negative breast cancer (38), and endometrial cancer (26). In this study, we used multiple HCC model cells to ascertain that SMIP34 diminished PELP1 expression and decreased cell viability and colony formation. Prior research demonstrated that the interaction between PELP1 and mTOR resulted in the activation of mTOR downstream signaling (39). Treatment of HCC cells with the PELP1 inhibitor SMIP34 effectively decreased the activity of mTOR and its downstream effectors, such as S6 and 4E-BP1. The findings from our investigation, utilizing SMIP34, are consistent with previously published research that establishes the involvement of PELP1 in mTOR signaling.

The dysregulation of PELP1 is a contributing factor to the development of therapy resistance (40) and metastases (11). Administering *PELP1*-siRNA liposomes to xenograft tumors resulted in a substantial decrease in tumor volume (41). Therapies specifically targeting PELP1 improved the effectiveness of chemotherapy (40). PELP1 signaling contributes to medulloblastoma progression by regulating the NF-κB pathway (42). It has been demonstrated that the modulation of metabolic PFKFB kinases by the PELP1/SRC-3 complex contributes to the development of therapy resistance (43). In this study, we have shown that KD of PELP1 or its pharmacologic inhibition using SMIP34 significantly reduces the progression of HCC *in vivo*. Considering the function of PELP1 in the promotion of chemotherapy resistance, future research is needed to assess the efficacy of combining SMIP34 with standard-of-care therapies for HCC, such as sorafenib. Despite the efficacy of SMIP34 *in vivo*, its translation to humans necessitates additional optimization of its potency to nanomolar and improved pharmacokinetic properties. Our ongoing research is focused on the optimization of SMIP34 using structure–activity relationship studies.

### Conclusions

In conclusion, our data provide the first evidence that the proto-oncogene PELP1 plays a critical role in the progression of HCC and that PELP1 suppression decreases HCC cell growth both *in vitro* and *in vivo*. Our results suggest that drugs targeting PELP1, such as SMIP34, may be useful as novel targeted therapy for treating HCC.

## Supplementary Material

Supplementary Table 1 Supplementary Table 1. List of all the primers used for RT-qPCR.

Supplementary Figure 1 Figure S1. Levels of PELP1 expression in 6 HCC cell lines.

Supplementary Figure 2 Figure S2. SMIP34 treatment downregulates liver specific genes, MYC and E2F pathway targeted genes in HCC cells.
