# Supplementary material for: PELP1 Is a Novel Therapeutic Target in Hepatocellular Carcinoma
Source: Cancer Res Commun. 2024 Oct 7;4(10):2610–20. doi: 10.1158/2767-9764.CRC-24-0173 (PMC11456993; doi:10.1158/2767-9764.CRC-24-0173)
Supplement: Supplementary Table 1 — List of all the primers used for RT-qPCR. [file crc-24-0173_supplementary_table_1_suppst1.pdf]

Supplementary Table 1. List of all the primers used for RT-qPCR

Gene list	Primers
CCNE1-F	5- AAGGAGCGGGACACCATGA -3
CCNE1-R	5- ACGGTCACGTTTGCCTTCC -3
BRCA2-F	5- TCAAGAGTTGGTTCTACGCTTC -3
BRCA2-R	5- CATGGGCAGAGCGATCTGT -3
MCM3-F	5- TCAGAGAGATTACCTGGACTTCC -3
MCM3-R	5- TCAGCCGGTATTGGTTGTCAC -3
BIRC5-F	5- AGGACCACCGCATCTCTACAT -3
BIRC5-R	5- AAGTCTGGCTCGTTCTCAGTG -3
POLA2-F	5- TCTTCGGCCTAGACTGCGA -3
POLA2-R	5- CTATGCCTGGCTTTCGATAATCT -3
TAF4B-F	5- CGCCCCTCCTAAAGTCAGC -3
TAF4B-R	5- TACCAACATCAACGGACCACT -3
HLF-F	5- CCACCTTTATCCCGCCTCC -3
HLF-R	5- TTTACTAAATGCGTCTTCGTGGT -3
CDCA7-F	5- GGGTGGCGATGAAGTTTCCA -3
CDCA7-R	5- GGGGATGTCTTCCACGGAAC -3
SAC3D1-F	5- AAGCCCTGCATGAGGTTCTAC -3
SAC3D1-R	5- CACTGCACAGCGCAACTTG -3
AZGP1-F	5- AACCAAGATGGTCGTTACTCTCT -3
AZGP1-R	5- CCTGCTTCCAATCCTCCATTC -3
AGTR1-F	5- ATTTAGCACTGGCTGACTTATGC -3
AGTR1-R	5- CAGCGGTATTCCATAGCTGTG -3
TM4SF4-F	5- AGGAAGCGGTGTCTTGATGAT -3
TM4SF4-R	5- GGAGGTGAACATCGCAAATCG -3
APOA2-F	5- GGAGCCATGTGTGGAGAGC -3
APOA2-R	5- CAGTTCCGTTCCAGCCTTCT -3
GHR-F	5- CCATTGCCCTCAACTGGACTT -3
GHR-R	5- AATATCTGCATTGCGTGGTGC -3
18S-F	5-GCTTAATTTGACTCAACACGGGA -3
18S-R	5-AGCTATCAATCTGTCAATCCTGTC -3
GAPDH-F	5- TCGACAGTCAGCCGCATCT-3
GAPDH-R	5- CTAGCCTCCCGGGTTTCTCT-3
