# Supplementary material for: PELP1 Is a Novel Therapeutic Target in Hepatocellular Carcinoma
Source: Cancer Res Commun. 2024 Oct 7;4(10):2610–20. doi: 10.1158/2767-9764.CRC-24-0173 (PMC11456993; doi:10.1158/2767-9764.CRC-24-0173)
Supplement: Supplementary Figure 1 — Figure S1. Levels of PELP1 expression in 6 HCC cell lines. [file crc-24-0173_supplementary_figure_1_suppsf1.pdf]

Figure S1

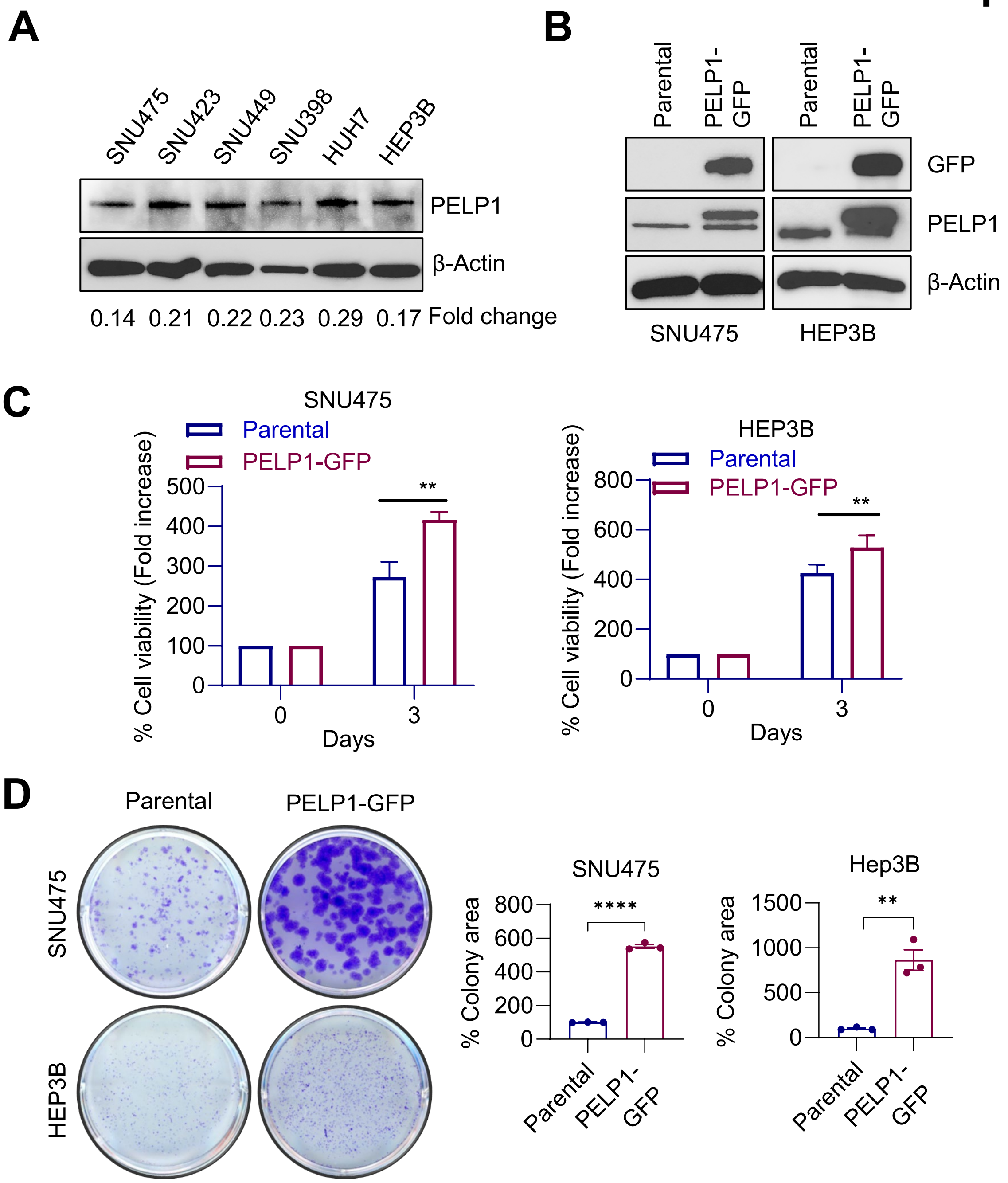

Figure S1. Levels of PELP1 expression in 6 HCC cell lines (A). PELP1 was overexpressed in SNU475 and Hep3B cell lines and expression was validated using GFP and PELP1 antibodies (B). PELP1 overexpression significantly increased cells proliferation (C) and colony formation (D) in HCC cell lines. Data are represented as mean ± SEM. *P* values are calculated using t test and two-way ANOVA, $^{**}p < 0.01$; $^{****}p < 0.0001$.
