# Supplementary material for: PELP1 Is a Novel Therapeutic Target in Hepatocellular Carcinoma
Source: Cancer Res Commun. 2024 Oct 7;4(10):2610–20. doi: 10.1158/2767-9764.CRC-24-0173 (PMC11456993; doi:10.1158/2767-9764.CRC-24-0173)
Supplement: Supplementary Figure 2 — Figure S2. SMIP34 treatment downregulates liver specific genes, MYC and E2F pathway targeted genes in HCC cells. [file crc-24-0173_supplementary_figure_2_suppsf2.pdf]

Figure S2

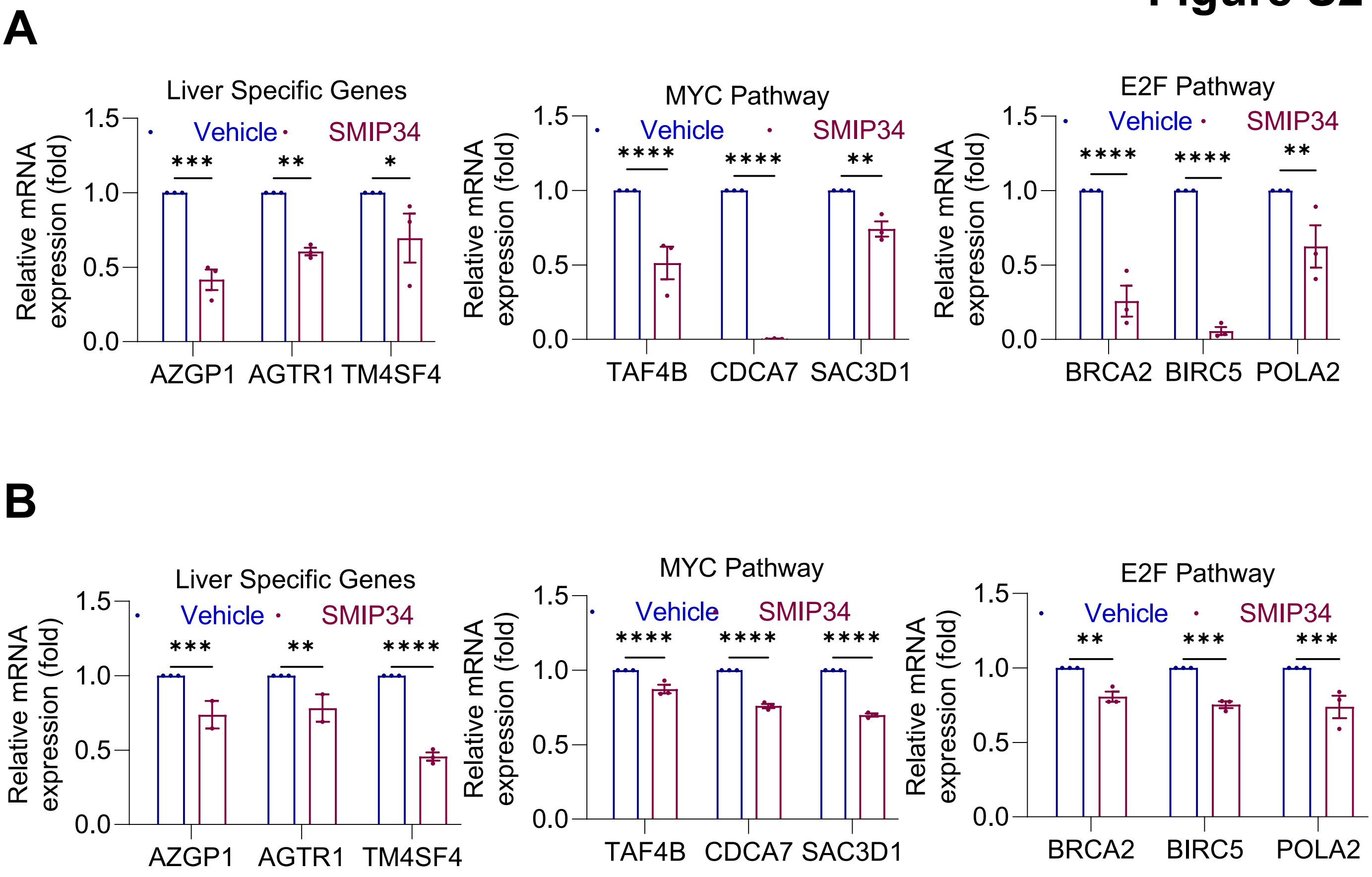

Figure S2. SMIP34 treatment downregulates liver specific genes, MYC and E2F pathway targeted genes in HCC cells. Hep3B (A) and SNU449 (B) cells were treated with either the vehicle or SMIP34 (12.5 μM) for 24 h, and RT-qPCR was used to validate the specific genes that were differentially regulated by PELP1-KD in RNA-seq analysis. Data are represented as mean ± SEM. *P* values are calculated using two-way ANOVA, *$p < 0.05$; **$p < 0.01$; ***$p < 0.001$; ****$p < 0.0001$.
